# A review collection on immunometabolism

**DOI:** 10.1093/lifemeta/load030

**Published:** 2023-07-02

**Authors:** Ping-Chih Ho, Chenqi Xu, Tiffany Horng

**Affiliations:** Department of Fundamental Oncology, University of Lausanne, 1007 Lausanne, Switzerland; Ludwig Institute for Cancer Research, University of Lausanne, 1066 Epalinges, Switzerland; State Key Laboratory of Molecular Biology, Shanghai Science Research Center, CAS Center for Excellence in Molecular Cell Science, Shanghai Institute of Biochemistry and Cell Biology, Chinese Academy of Sciences, Shanghai 200031, China; School of Life Sciences and Technology, ShanghaiTech University, Shanghai 201210, China

Recent studies indicate an intimate link between immunity and metabolism, spawning the now burgeoning field of immunometabolism. What is the rationale for such a link? On the one hand, immune responses are energetically very demanding. Clonal expansion of T and B cells, increased production of ­inflammatory cytokines and antibodies by activated macrophages and plasma cells, and recruitment of immune cells to the site of infection are all thought to impose a high metabolic and energetic demand. This may explain why cellular metabolism regulates the ­activation/differentiation and proliferation of T and B cells, as well as the effector functions of all immune cells. On the other hand, immune cells play a fundamental role in regulating metabolism and homeostasis. Maintenance of homeostasis is ­necessary to life and health, and in many respects, metabolism can be considered a first line of defense in such maintenance, for example, sustaining nutrient and energy homeostasis in response to nutrient influx and nutrient deprivation. Immunity can be considered a second line of defense in the maintenance of homeostasis and is called into action when metabolism is insufficient to do the job. For example, if adipocytes are not able to handle nutrient surplus or deficiency in a cell-autonomous manner, ­adipose ­tissue-resident immune cells can be engaged to help adipocytes do so. Therefore, immunity and metabolism are inextricably linked in coordinating homeostasis and health, while dysregulation of such a link predisposes to chronic diseases, many of which are the most common diseases of the 21st century.

In this review collection, *Life Metabolism* commissioned four reviews that cover both metabolic support of immunity and immune cell regulation of homeostasis and metabolism. In the first review, Peng and Li [1] discuss the influence of cellular metabolism throughout the life journey of T lymphocytes and introduce the concept that different metabolic pathways (­glycolysis, lipid metabolism, etc.) can regulate various T-cell fate decisions as well as T effector functions. In the second review, Ke *et al.* [2] focus on the role of lipid metabolism, including fatty acid and cholesterol metabolism, in regulating T-cell biology. Another focus of this review is on how the hostile conditions of the tumor microenvironment enforce changes to the lipid metabolism of infiltrating T cells to modulate their antitumor activities, and whether this process can be targeted in cancer immunotherapy. The third review in this collection from Sun *et al.* [3] summarizes how circadian metabolism influences rhythmicity in macrophage elaboration of inflammatory responses and other macrophage activities. Circadian metabolism refers to the rhythmic nature of metabolism over the course of day–night cycles, and this review discusses how in macrophages, such rhythmicity contributes to time-of-day dependent changes in inflammatory responses, allowing for maximization of host defense yet minimization of inflammation-associated tissue damage. Finally, the last review from Bevilacqua *et al.* [4] presents an overview of how cellular metabolism in T cells is altered in aging to contribute to reduced T-cell functionality. The consequences of such metabolic alterations to inflammaging, which is linked to age-associated loss of tissue homeostasis and tissue function, are also considered.

In summary, this review collection focuses on four timely aspects of the fundamental connection between immunity and metabolism. We hope that these reviews will stimulate discussion and look forward to many more studies in the vibrant field of immunometabolism.
